# A general framework for quantifying the effects of DNA repair inhibitors on radiation sensitivity as a function of dose

**DOI:** 10.1186/1742-4682-4-25

**Published:** 2007-07-19

**Authors:** Anthony J Chalmers, Soeren M Bentzen, Francesca M Buffa

**Affiliations:** 1 Brighton and Sussex Medical School, University of Sussex, Falmer, Brighton BN1 9RQ, UK; 2 University of Wisconsin Medical School, Department of Human Oncology, K4/316 Clinical Sciences Center, 600 Highland Avenue, Madison, WI 53792, USA; 3 Cancer Research UK Molecular Oncology Laboratories, Weatherall Institute of Molecular Medicine, University of Oxford, John RadcliffeHospital, Oxford OX3 9DU, UK

## Abstract

**Purpose:**

Current methods for quantifying effects of DNA repair modifiers on radiation sensitivity assume a constant effect independent of the radiation dose received. The aim of this study was to develop and evaluate a modelling strategy by which radiation dose dependent effects of DNA repair inhibitors on clonogenic survival might be identified and their significance assessed.

**Methods:**

An indicator model that allowed quantification of the Sensitiser Effect on Radiation response as a function of Dose (SERD) was developed. This model was fitted to clonogenic survival data derived from human tumour and rodent fibroblast cell lines irradiated in the presence and absence of chemical inhibitors of poly(ADP-ribose) polymerase (PARP) activity.

**Results:**

PARP inhibition affected radiation response in a cell cycle and radiation dose dependent manner, and was also associated with significant radiation-independent effects on clonogenic survival. Application of the SERD method enabled identification of components of the radiation response that were significantly affected by PARP inhibition and indicated the magnitude of the effects on each component.

**Conclusion:**

The proposed approach improves on current methods of analysing effects of DNA repair modification on radiation response. Furthermore, it may be generalised to account for other parameters such as proliferation or dose rate to enable its use in the context of fractionated or continuous radiation exposures.

## Background

Radiotherapy is an effective mode of cancer treatment but its capacity to cure is limited by toxic effects on healthy tissues. Developing effective treatment schedules requires detailed knowledge of the cellular effects of radiation in tumours and normal tissues so that differences may be exploited and a beneficial therapeutic ratio achieved. Increasing evidence indicates that DNA repair pathways are a key determinant of cell survival after radiation, and that targeting the molecular components of these pathways offers therapeutic potential [1-3].

When assessing the impact of modifiers of DNA repair on cellular responses to ionising radiation, accurate measurement of effects on clonogenic survival is crucial, since this is the most clinically relevant radiation response [4]. Data are generally presented in the form of survival curves, which illustrate radiation effects over a range of doses and may be described by parameters that derive primarily from the Linear Quadratic (LQ) equation [5]. It is well established, however, that radiation sensitivity may deviate from the LQ model, especially at low doses; mathematical models have been generated to indicate the extent of such deviation [6]. Assessing the effect of DNA repair modification on the whole dose-response curve represents an additional challenge that must be overcome if accurate assessment of the biological consequences and therapeutic potential of DNA repair modifiers is to be achieved.

A conventional approach is to calculate a Sensitiser Enhancement Ratio (SER) from the radiation dose (D_SF_) associated with a specified surviving fraction, typically 37% (D_0_)[7], or from the surviving fraction associated with a specified radiation dose, typically 2 Gray (SF_2_)[8]:

SER=D0 without sensitiserD0 with sensitiserorSER=SF2 without sensitiserSF2 with sensitiser
 MathType@MTEF@5@5@+=feaafiart1ev1aaatCvAUfKttLearuWrP9MDH5MBPbIqV92AaeXatLxBI9gBaebbnrfifHhDYfgasaacH8akY=wiFfYdH8Gipec8Eeeu0xXdbba9frFj0=OqFfea0dXdd9vqai=hGuQ8kuc9pgc9s8qqaq=dirpe0xb9q8qiLsFr0=vr0=vr0dc8meaabaqaciaacaGaaeqabaqabeGadaaakeaafaqabeqadaaabaGaee4uamLaeeyrauKaeeOuaiLaeyypa0tbaeaabiqaeaqaaiabbseaenaaBaaaleaacqaIWaamaeqaaOGaeeiiaaIaee4DaCNaeeyAaKMaeeiDaqNaeeiAaGMaee4Ba8MaeeyDauNaeeiDaqNaeeiiaaIaee4CamNaeeyzauMaeeOBa4Maee4CamNaeeyAaKMaeeiDaqNaeeyAaKMaee4CamNaeeyzauMaeeOCaihabaGaeeiraq0aaSbaaSqaaiabicdaWaqabaGccqqGGaaicqqG3bWDcqqGPbqAcqqG0baDcqqGObaAcqqGGaaicqqGZbWCcqqGLbqzcqqGUbGBcqqGZbWCcqqGPbqAcqqG0baDcqqGPbqAcqqGZbWCcqqGLbqzcqqGYbGCaaaabaGaem4Ba8MaemOCaihabaGaee4uamLaeeyrauKaeeOuaiLaeyypa0tbaeaabiqaeaqaaiabbofatjabbAeagnaaBaaaleaacqaIYaGmaeqaaOGaeeiiaaIaee4DaCNaeeyAaKMaeeiDaqNaeeiAaGMaee4Ba8MaeeyDauNaeeiDaqNaeeiiaaIaee4CamNaeeyzauMaeeOBa4Maee4CamNaeeyAaKMaeeiDaqNaeeyAaKMaee4CamNaeeyzauMaeeOCaihabaGaee4uamLaeeOray0aaSbaaSqaaiabikdaYaqabaGccqqGGaaicqqG3bWDcqqGPbqAcqqG0baDcqqGObaAcqqGGaaicqqGZbWCcqqGLbqzcqqGUbGBcqqGZbWCcqqGPbqAcqqG0baDcqqGPbqAcqqGZbWCcqqGLbqzcqqGYbGCaaaaaaaa@A035@

SER values calculated in this way reflect the impact of a repair modifier at a single dose or survival point [9-11]. D_0 _and SF_2 _may also be estimated by fitting survival data as a function of dose [12], thus reflecting the whole data set, but neither method has the capacity to quantify differential sensitising effects over different radiation dose ranges.

Another approach is to calculate the effect of a modifier on the *α *and *β *parameters of the LQ equation, which describe respectively the linear and exponential components of the survival curve [12,13]. Ratios calculated from these parameters give an indication of both magnitude and radiation dose dependency of a sensitising effect, but the method has limitations.

Firstly, the fitting of these models has always been performed separately on the treated and untreated datasets, making direct comparison of the parameters difficult. Furthermore, it cannot be applied in situations where the relationship between survival and dose is more complex than that predicted by the LQ equation. Secondly, cytotoxic effects of sensitising agents that are independent of radiation are not taken into account. Such effects may be small, and are often concealed by the method used to calculate surviving fraction, but may be relevant, particularly if they vary between cell lines or are of similar magnitude to the radiation modifying effects under investigation. In such cases, it would be informative to assess the relative significance of the cytotoxic and radiosensitising effects, and to ascertain whether the two are interdependent.

The aim of this project was to devise a general approach that could be applied to complex survival curves and used to quantify: (1) drug-induced changes in survival at different radiation doses, (2) radiation-independent effects on survival and (3) the relative significance of these changes. The main features of the method are the inclusion of an indicator term in the model to indicate the presence of the drug and a factor *δ*x representing the variation on any parameter of survival between radiation only and radiation plus drug. This implies that the perturbation on the parameters introduced by the drug can be approximated using linear regression and that the linear regression can be truncated to the first term. The first of these assumptions is quite general as a large variety of problems can be treated within a linear regression framework; the second holds only if the perturbation is linear or relatively small. However, the model can easily be extended to include higher order linear regression terms.

In this study the LQ equation was modified using Joiner's Induced Repair model [6] and used to express survival at a given dose, but the approach is general and may be applied to any other expression of survival. To test the applicability of the approach, the model was fitted to a range of clonogenic survival curves that had been previously derived from rodent and human cell lines irradiated in the presence and absence of two chemical inhibitors of the DNA repair enzyme poly(ADP-ribose) polymerase (PARP).

## Results

### Development of model: Sensitiser Effect on Radiation response as a function of Dose (SERD)

Log transformed surviving fraction, *SF*, was fitted as a function of dose, *d*, using the indicator model:

SF(d)=(1+δz⋅i)⋅exp⁡ {−(α+δα⋅i)⋅d−(G+δG⋅i)⋅e−d/(dC+δdc⋅i)⋅d−(β+δβ⋅i)⋅d2}
 MathType@MTEF@5@5@+=feaafiart1ev1aaatCvAUfKttLearuWrP9MDH5MBPbIqV92AaeXatLxBI9gBaebbnrfifHhDYfgasaacH8akY=wiFfYdH8Gipec8Eeeu0xXdbba9frFj0=OqFfea0dXdd9vqai=hGuQ8kuc9pgc9s8qqaq=dirpe0xb9q8qiLsFr0=vr0=vr0dc8meaabaqaciaacaGaaeqabaqabeGadaaakeaacqWGtbWucqWGgbGrcqGGOaakcqWGKbazcqGGPaqkcqGH9aqpcqGGOaakcqaIXaqmcqGHRaWkiiGacqWF0oazcqWG6bGEcqGHflY1cqWGPbqAcqGGPaqkcqGHflY1cyGGLbqzcqGG4baEcqGGWbaCcqqGGaaidaGadaqaaiabgkHiTiabcIcaOiab=f7aHjabgUcaRiab=r7aKjab=f7aHjabgwSixlabdMgaPjabcMcaPiabgwSixlabdsgaKjabgkHiTiabcIcaOiabdEeahjabgUcaRiab=r7aKjabdEeahjabgwSixlabdMgaPjabcMcaPiabgwSixlabdwgaLnaaCaaaleqabaGaeyOeI0IaemizaqMaei4la8IaeiikaGIaemizaq2aaSbaaWqaaiabdoeadbqabaWccqGHRaWkcqWF0oazcqWGKbazdaWgaaadbaGaem4yamgabeaaliabgwSixlabdMgaPjabcMcaPaaakiabgwSixlabdsgaKjabgkHiTiabcIcaOiab=j7aIjabgUcaRiab=r7aKjab=j7aIjabgwSixlabdMgaPjabcMcaPiabgwSixlabdsgaKnaaCaaaleqabaGaeGOmaidaaaGccaGL7bGaayzFaaaaaa@89E8@

where *δz *allows for non-null effect of the drug on plating efficiency; *α *and *β *are the classical linear and quadratic radiosensitivity parameters; *G *and *d*_C _are the low-dose hyper-sensitivity parameters [14]; *i *is an indicator which assumes the value zero for the control case, i.e. radiation alone, and one for the drug-treated case; and *δ*x – where "x" is any of the parameters above – is the variation on x between the control and case under study. General least square fitting was used and the significance of terms in the model was tested using the log-likelihood ratio test. This test considers the ratio of the likelihood of the model with the parameter to the model without the parameter. Terms which showed non-significant improvement were removed from the model; terms which gave a *p*-value of < 0.05 were considered significant and retained in the final model (see Table 1). Retention of a *δ*x parameter in the final model thus indicated a significant drug effect. S-PLUS 6.1 was used for implementation of the methods and the analysis [15].

**Table 1:** Significant coefficients generated by fitting the SERD equation to the survival curves shown in Figures 1, 2 and 3.

Cell line	Parameter	Value (± standard error)	*p*-value*
CHO-K1 (Fig 1a)	*α*	0.142 (± 0.021)	<0.0001
	*β*	0.043 (± 0.005)	<0.0001
	*δz*	-0.133 (± 0.023)	<0.0001
	*δα*	0.112 (± 0.015)	<0.0001
	*δG*	34.649 (± 12.328)	0.005
	*δd*_C_	0.037 (± 0.008)	<0.0001
V79-379A (Fig 1b)	*α*	0.187 (± 0.019)	<0.0001
	*β*	0.016 (± 0.004)	0.0003
	*G*	2.235 (± 0.666)	0.0009
	*d*_C_	0.161 (± 0.031)	<0.0001
	*δz*	-0.184 (± 0.017)	<0.0001
T98G exponential phase (Fig 2a)	*α*	0.208 (± 0.006)	<0.0001
	*δz*	-0.101 (± 0.014)	<0.0001
	*δG*	10.116 (± 10.374)	0.330
	*δG*	7.81	0.020
	*δd*_C_	0.033 (± 0.019)	0.076
	*δβ*	0.013 (± 0.002)	<0.0001
T98G growth-arrested (Fig 2b)	*α*	0.175 (± 0.003)	<0.0001
	*δz*	0.051 (± 0.007)	<0.0001
	*δα*	-0.017 (± 0.005)	0.0005
U373-MG exponential phase (Fig 3a)	*α*	0.270 (± 0.011)	<0.0001
	*δz*	0.068 (± 0.021)	0.002
	*δβ*	0.028 (± 0.004)	<0.0001
U373-MG growth-arrested (Fig 3b)	*α*	0.126 (± 0.014)	<0.0001
	*β*	0.031 (± 0.003)	<0.0001
	*δz*	-0.044 (± 0.012)	0.0002

* Log-likelihood ratio test (L-ratio) was applied to include or drop parameters from the final equation. *p*-values shown were derived from a t-test that the parameter is zero. The L-ratio and associated *p*-value is shown only when the tests did not agree (i.e. significance in one but not the other).

In Joiner's original paper, the low-dose hypersensitivity parameter *g *was defined as: *g *= (*α*_*S *_- *α*_*R*_)/*α*_*R *_where *α*_*S *_is derived from the very low dose component of the survival curve and *α*_*R *_from the overall linear component of the curve. To reduce correlation between the model variables and to facilitate implementation of the model, we have used here a re-parameterisation of the model where G = α_S _- α_R_.

In the indicator model (Equation 1), the radiosensitivity parameter change for drug-treated cells is in the form x+*δ*x, the underlying hypothesis being that the perturbation introduced by the drug effect on the radiation parameters can be approximated to its linear component in the first instance. For prolonged or fractionated irradiation regimes, parameters associated with repopulation or repair effects could also be incorporated into the model.

Although a degree of correlation between the various parameters in Equation 1 could be expected, this method allows quantification of linear, quadratic and low dose survival, and direct comparison of these parameters between control and drug-treated cells.

### Evaluation of SERD model

**Figure 1:**
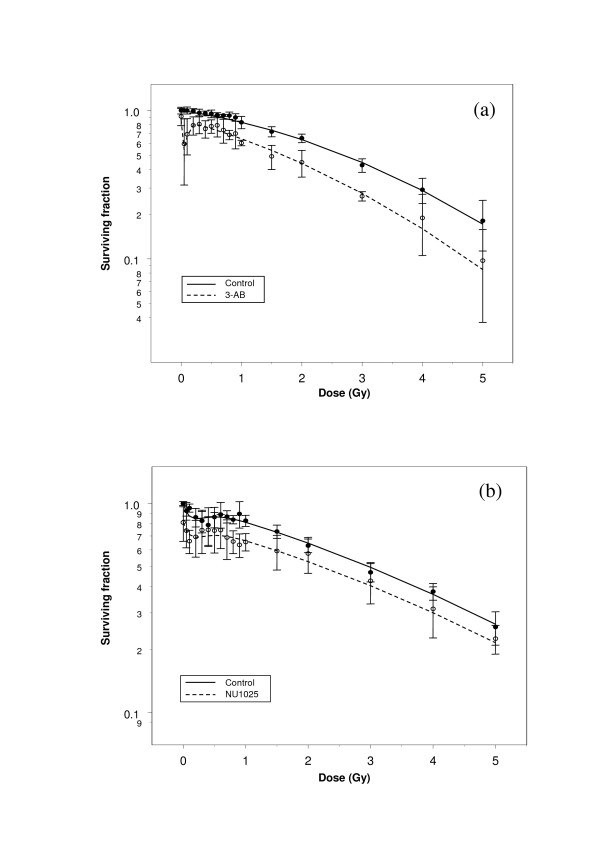
Clonogenic survival curves derived from asynchronous, irradiated populations of (a) CHO-K1 hamster fibroblasts +/- 5 mM 3-aminobenzamide and (b) V79-379A hamster fibroblasts +/- 100 μM NU1025. In all figures, data points represent means (+/- standard error of the mean) of three independent experiments.

**Figure 2:**
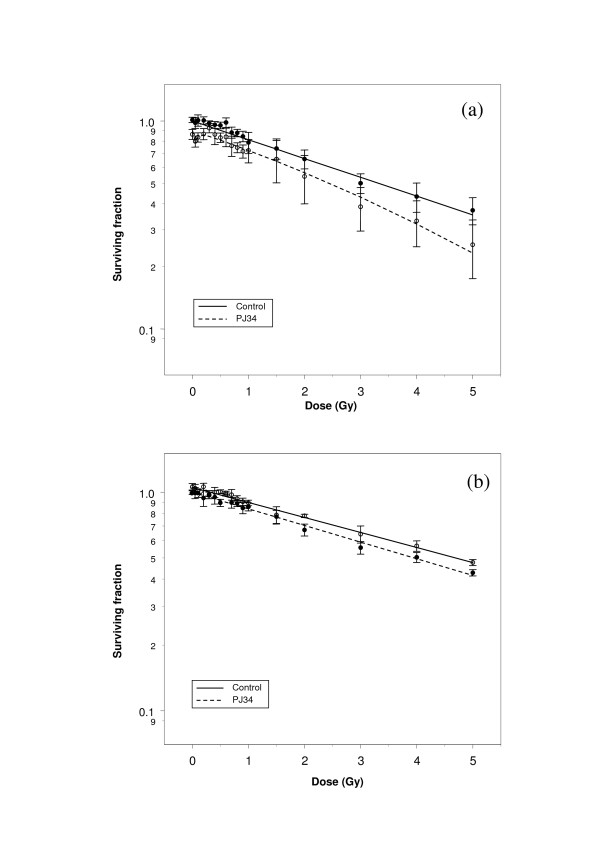
Clonogenic survival curves derived from (a) exponential and (b) confluence-arrested populations of T98G glioma cells irradiated +/- 3 μM PJ34.

**Figure 3:**
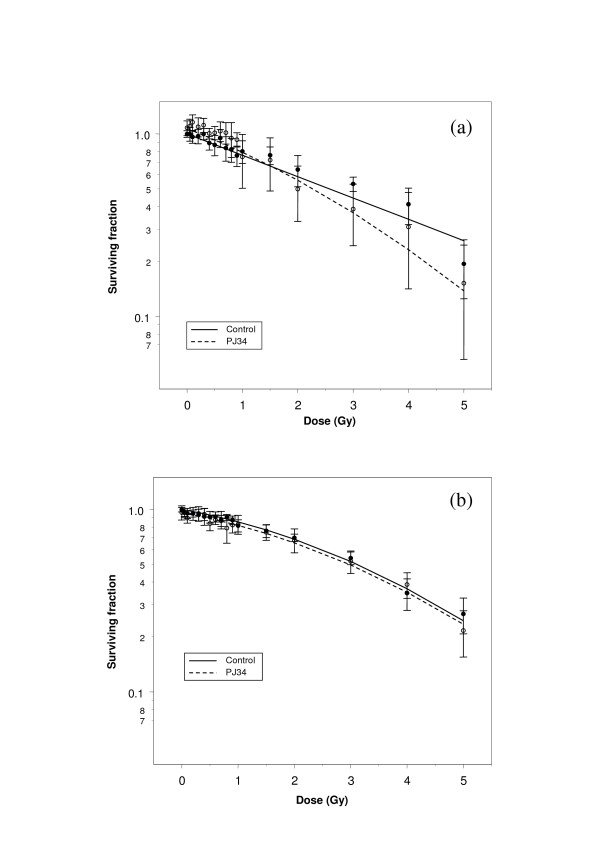
Clonogenic survival curves derived from (a) exponential and (b) confluence-arrested populations of U373-MG glioma cells irradiated +/- 3 μM PJ34.

Figures 1, 2, 3 show clonogenic survival curves generated by irradiation of two rodent fibroblast and two human tumour cell lines in the presence and absence of chemical inhibitors of PARP activity. The variable nature and magnitude of the effects of PARP inhibition on clonogenic survival among these cell lines offered a useful setting in which to investigate the utility and applicability of the SERD model. The radiobiological implications of the curves have been published elsewhere [16] and will not be discussed here.

The survival curves shown in figure 1a illustrate radiosensitisation of CHO-K1 fibroblasts by 3-AB, with marked effect over the dose range 0.05 – 0.3 Gy. Fitting the model in equation 1 to these data demonstrated that the control curve is described by the classic linear quadratic equation, with *α *and *β *emerging as the only significant parameters, while the high value derived for *δG *denoted a significant effect of 3-AB on low-dose hyper-radiosensitivity (Table 1). Addition of the drug also exerted a negative effect on radiation-independent survival (*δz *significant and retained in the reduced model), and enhanced the linear (*δα *significant) but not the quadratic component of cell killing (*δβ *non-significant).

Similar analysis of the curves in figure 1b indicated that V79-379A cells exhibited significant low-dose hyper-radiosensitivity in the absence of PARP inhibitor, and that radiation-independent survival was significantly reduced in its presence. No significant interaction between NU1025 and any parameter of radiosensitivity was identified.

Figure 2a illustrates modification of the low-dose survival characteristics of exponential phase T98G glioma cells by PJ34. Fitting the SERD equation to these data indicated that the effect of the drug on the low-dose hyper-radiosensitivity parameter *G *was modest and did not reach statistical significance. However, the fit of the model was significantly superior when the *δG *parameter was included than when it was not (see log likelihood ratio); thus it was retained in the reduced final model after likelihood testing. This supports the interpretation that PJ34 induces low-dose hyper-radiosensitivity in exponential phase populations of T98G.

By contrast, analysis of figures 2b, 3a and 3b indicated that PJ34 did not affect low-dose radiation sensitivity of confluent populations of T98G glioma cells, or of U373-MG cells. In all cases, the radiation-independent effect of the drug on survival (*δz*) was a significant parameter.

The effect of PJ34 on overall radiosensitivity of human glioma cells was dependent on the cell cycle characteristics of the irradiated population. In exponential phase populations, addition of the drug increased the quadratic component of cell killing (Figs. 2a, 3a), whereas in growth-arrested populations there was no radio-sensitisation (Figs. 2b, 3b). The negative effect of PJ34 on the linear component of cell killing in growth-arrested T98G cells may reflect a modest radioprotective effect of the drug in this population.

## Discussion

Conventional analysis of the effects of DNA repair modifiers upon clonogenic survival is limited to quantifying the magnitude of change of a single survival parameter, typically D_0 _or SF_2_. This approach fails to take into account dose-dependent variations in response modification, and is unsuited to the analysis of complex or multiphasic survival curves. Furthermore, many modifiers exert a radiation-independent effect on survival that renders interpretation of their impact on the low dose region of the survival curve problematic. Finally, as fitting of the model is usually performed separately on treated and untreated survival curves, the parameters are not directly comparable. The SERD method presented here was generated to enable direct comparison of the parameters in the treated and untreated experiments. As a consequence, quantitative assessment of the effect of modifiers of DNA repair upon four distinct components of the radiation response was achieved: (1) radiation-independent survival (parameter *z*, Equation 1), (2) low-dose radiation sensitivity (parameters *G *and *d*_*c*_), (3) the linear component of cell survival (*α*), and (4) the quadratic component of cell survival (*β*). A data set comprising complex survival curves and varied responses to DNA repair modification was used to test the applicability of the SERD equation.

In the absence of an existing method by which survival parameters can be directly compared between treated and untreated experiments, the merits of the approach were evaluated in terms of the capacity of the model to quantify and indicate the relative significance of the effects of PARP inhibition on the survival parameters listed above. On a more subjective level, the ability of the model to enhance interpretation of complex survival data was considered.

Application of the SERD equation to the data derived from hamster fibroblast cell lines indicated that, while 3-AB significantly affected radiosensitivity parameters in CHO-K1 fibroblasts, any radiosensitising effects of NU1025 in V79-379A fibroblasts were rendered non-significant by the radiation-independent effect of the drug. Inclusion in the model of the radiation-independent parameter *z *thus enabled more robust assessment of drug effects. The model also indicated that radiosensitising effects of 3-AB on CHO-K1 cells were restricted to linear and low dose hypersensitivity parameters.

When applied to data derived from human glioma cell lines, the method was shown to be sensitive to subtle changes in shape and gradient of survival curves. An effect of PARP inhibition on low-dose sensitivity of exponential phase T98G cells was substantiated by the SERD model, but the magnitude of the effect was demonstrably smaller than in CHO-K1 cells. Likewise, diverse effects of PARP inhibition on exponential phase and growth-arrested populations of glioma cells were validated by the model.

The observation that *δz *was a significant parameter in all cases, and that the magnitude and direction of this effect varied according to cell line and confluence, suggests that this variable is an important factor in the measurement of radiation responses. Including *δz *in the SERD equation enabled investigation of its relationship with radiation-dependent parameters; other methods require correction for radiation-independent effects prior to analysis.

## Conclusion

Measurement of radiation responses over a wide range of doses is becoming increasingly accurate [17], and examples of radiation dose-dependent mechanisms are emerging [18,19]. In its current form, we have shown the SERD method to be a useful tool in the analysis of survival data that are not adequately described by the linear quadratic equation, and in the evaluation of modifiers of the radiation response. Since the framework chosen allows direct comparison of all new parameters considered, additional parameters could be incorporated into the model in a structured way to facilitate its application to scenarios in which additional radiobiological phenomena such as repair or repopulation might be important.

## Methods

### Cell lines and chemical inhibitors

T98G and U373-MG human glioblastoma cells and CHO-K1 and V79-379A hamster fibroblast cells were routinely maintained in monolayer culture in Eagle's minimal essential medium supplemented with 10% fetal calf serum. For experiments using growth-arrested populations, cells were allowed to reach confluence and harvested 24 h later, after discarding detached cells. For all other experiments, exponentially growing cells were harvested at 50% confluence. 3-aminobenzamide (3-AB) (Sigma-Aldrich, Dorset), PJ34 (Calbiochem), and NU1025 (generous gift of Dr. B Durkacz of Newcastle University) were administered in tissue culture medium warmed to 37°C at concentrations determined in preliminary cytotoxicity assays: 5 mM 3-AB, 100 μM NU1025 and 3 μM PJ34.

### Clonogenic survival assay

Clonogenic survival assays were carried out using the flow cytometric cell-sorting protocol described previously [16]. Briefly, precise numbers of cells were plated by flow cytometric sorting and incubated for 2 hours for adherence. Medium was then replaced with prewarmed control or drug-containing medium. Flasks were irradiated (0.05 – 5 Gy) with 240 kV X-rays after a further 2 hours and drug-free medium replaced 22 hours later. After an incubation period of seven cell doubling times, surviving colonies were stained with crystal violet solution and counted.

Each plot was derived from a minimum of three independent experiments, each performed in triplicate. Plating efficiencies were calculated for all flasks, and surviving fraction for drug-free flasks was calculated in the usual way. For drug-treated flasks, surviving fraction was calculated using the mean, unirradiated, drug-free plating efficiency as the denominator. This method revealed radiation-independent drug effects and enabled assessment of the relationship of this variable to radiation-dependent effects.

## Competing interests

The author(s) declare that they have no competing interests.

## Authors' contributions

AC participated in the design of the study, executed the laboratory experiments and drafted the manuscript. SB participated in the design of the study and advised on statistical methodology. FB participated in the design of the study, developed and performed the statistical analysis and helped to draft the manuscript. All authors read and approved the final manuscript.
